# Administration sequences in single-day chemotherapy regimens for breast cancer: a comprehensive review from a practical perspective

**DOI:** 10.3389/fonc.2024.1353067

**Published:** 2024-09-30

**Authors:** Miaohui Wu, Xiaoyan Huang, Meijun Chen, Yin Zhang

**Affiliations:** ^1^ Department of Pharmacy, The Second Affiliated Hospital of Fujian Medical University, Quanzhou, China; ^2^ Department of Endocrinology, Quanzhou First Hospital Affiliated to Fujian Medical University, Quanzhou, China; ^3^ Department of Pharmacy, Clinical Oncology School of Fujian Medical University & Fujian Cancer Hospital, Fuzhou, China

**Keywords:** breast cancer, chemotherapy, sequence, single-day, review

## Abstract

**Introduction:**

Breast cancer is one of the most prevalent malignant tumors globally, posing a severe threat to human life and health. Chemotherapy, a cornerstone in the treatment of breast cancer, often overlooks the sequence of drug administration within single-day regimens. This study aims to explore the impact of drug administration order on the efficacy and toxicity of combination chemotherapy protocols for breast cancer.

**Methods:**

Through a comprehensive review and analysis based on current evidence from evidence-based medicine, we delved into how the order of drug administration affects both efficacy and toxicity. We systematically classified and analyzed commonly used combination drug regimens, providing graded recommendations and a reasoned analysis to offer valuable references for clinical decision-making.

**Results:**

Our findings indicate that the sequence of drug administration in complex combination chemotherapy protocols is not arbitrary but necessitates multifaceted considerations. Rational drug sequencing can maximize synergistic effects between drugs, thereby augmenting therapeutic efficacy while effectively mitigating drug-related adverse effects. Additionally, some drug labels and clinical trials have explicitly highlighted the therapeutic benefits of specific drug sequences.

**Conclusion:**

This study underscores the importance of considering the sequence of drug administration in clinical practice. It is recommended to prioritize the sequential drug administration according to official drug product labeling, while also considering factors such as the administration sequence from large randomized controlled trials, cell proliferation kinetics specific to cancer types, drug interactions, chronopharmacology, drug irritability, clinical experiences, and patient preferences. By taking these factors into account, the goal is to maximize treatment efficacy and minimize the occurrence of adverse reactions.

## 1 Introduction

Breast cancer, as one of the most prevalent malignancies globally, poses a severe threat to human life and health, ranking as the leading cause of female mortality (1). In the protracted battle against cancer, chemotherapy stands as a cornerstone of treatment, its significance undeniable.

Chemotherapeutic agents encompass a diverse range of drugs that eliminate or inhibit cancer cell growth through multiple mechanisms, extensively employed in breast cancer management. Based on their mechanisms of action and chemical structures, these agents are primarily categorized into five major classes: anthracyclines, platinum compounds, alkylating agents, taxanes, and antimetabolites. Anthracyclines, such as doxorubicin and epirubicin, inhibit cancer cell proliferation by intercalating into the DNA double helix, disrupting DNA replication and transcription. They are routinely administered in the treatment of HER2-negative breast cancer, particularly in high-risk patients (2, 3). Platinum compounds, including cisplatin and carboplatin, exhibit potent antitumor activity by forming adducts with DNA. They are not only used in the treatment of triple-negative breast cancer (TNBC) but also widely applied across various stages of breast cancer (4–6). Alkylating agents bind covalently to cancer cell DNA, halting DNA replication and transcription. Cyclophosphamide, for instance, is commonly utilized in adjuvant and neoadjuvant therapy for hormone receptor-positive breast cancer (7, 8). Taxanes stabilize microtubules, impeding mitosis during cell division and thereby inhibiting cancer cell proliferation. Drugs like paclitaxel and docetaxel are frequently prescribed for metastatic breast cancer and lymph node-positive disease (9). Additionally, antimetabolites disrupt DNA and RNA synthesis in cancer cells. Drugs such as fluorouracil and gemcitabine are commonly administered in neoadjuvant therapy for locally advanced breast cancer and in the management of metastatic breast cancer (10, 11).

Chemotherapeutic agents play a pivotal role in breast cancer treatment, inhibiting cancer cell growth and proliferation through diverse mechanisms. However, with the advancement of scientific research, monotherapy with a single agent often struggles to address the complex and heterogeneous nature of cancer. Consequently, optimizing chemotherapy regimens, particularly through the combination of multiple drugs to enhance therapeutic efficacy, improve patient tolerance, and mitigate drug resistance, has emerged as a research frontier in contemporary cancer therapy (12–15). Notably, the administration of multiple drugs on the same day has become commonplace in clinical practice; however, the sequence of drug delivery is often underappreciated or overlooked. This neglect stems from two primary sources: first, a pervasive misconception that the order of administration for different drugs has negligible impact on treatment outcomes; second, a paucity of research focusing on chemotherapy sequencing, particularly lacking in systematic analyses or series of studies based on big data.

In reality, the sequencing of drugs in complex combination chemotherapy protocols is not arbitrary but necessitates multifaceted considerations. In recent years, a growing body of research and treatment guidelines has emphasized the importance of specific drug administration sequences in combined chemotherapy regimens. These studies suggest that rational drug sequencing can maximize the synergistic effects between drugs, thereby augmenting therapeutic efficacy while effectively mitigating drug-related adverse effects (16, 17). Additionally, some drug labels and clinical trials have explicitly highlighted the therapeutic benefits of specific drug sequences, further substantiating the scientific validity and practical applicability of this notion (18).

Based on our preliminary investigation, while several studies have delved into the sequencing of chemotherapeutic drugs, they remain scattered, with disparate conclusions and inherent uncertainties. Notably, the research landscape concerning chemotherapy sequencing in breast cancer remains largely unexplored, directly impacting the standardization and personalized precision of chemotherapy regimens. Given this backdrop, our study aims to comprehensively collect and scrutinize relevant drug data, endeavoring to conduct a rational analysis of drug sequencing in combined chemotherapy protocols for breast cancer, thereby providing insights for future clinical treatment practices.

## 2 Materials and methods

### 2.1 Preliminary work

Prior to the collection of evidence-based data, we initially extracted commonly utilized chemotherapy regimens for breast cancer. This was achieved by thoroughly reviewing breast cancer diagnosis and treatment guidelines (19–21), The focus was on identifying combination chemotherapy regimens listed within these guidelines, specifically those involving the administration of two or more drugs within the span of a single day (24 hours).

### 2.2 Search strategy

We conducted a literature search, performed data extraction, and carried out a systematic review in accordance with the principles outlined in the PRISMA guidelines, we employed a search strategy with key terms. Queries included the MeSH terms “chemotherapy,” “sequence,” “immediately following,” “sequential,” “order,” “in turn,” “time,” “single day,” and “one day.” This strategy was used to retrieve relevant information on chemotherapy drug sequencing from diverse sources, including PubMed, Web of Science, The Cochrane Library, Embase, CNKI, PubMed, Wan Fang database and pertinent professional literature. In addition, we are also searching for drug registration documents in the databases of the U.S. FDA (U.S. FOOD & DRUG ADMINISTRATION) and CENTER FOR DRUG EVALUATION (NMPA).

### 2.3 Article selection

We conducted a search in databases up to October 2023 for original articles published since the inception of the database. These articles explored the advantages and disadvantages of relevant drug administration sequences. Excluded from the review were abstracts, patents, conference discussions, posters, and articles without full-text access.

### 2.4 Article screening

The data extracted from the articles includes drug regimens, administration sequences, and their merits and demerits, primarily focusing on efficacy and toxicity. With two trained pharmacists independently conducting the process. The subsequent compilation involved a third independent reviewer who conducted an audit of data uncertainties. Any contentious issues are thoughtfully resolved through collaborative discussions within the research team, ultimately determining the final dataset.

### 2.5 Article classification

This study utilized the Thomson^®^ grading system from Micromedex, integrating evidence quality and team discussions to categorize medication sequences into three tiers. Grade A comprises numerous, well-designed, and large-scale meta-analyses of randomized controlled trials (RCTs) that provide high-quality evidence, demonstrating robustness and safety. Therefore, we recommend the adoption of such medication sequences. Grade B includes meta-analyses of RCTs with conflicting conclusions, as well as small-scale studies or those with some methodological flaws, and RCTs carrying a certain risk of bias, along with non-randomized studies. Despite lacking some validation and robust evidence, medication sequences falling into this category can still serve as references. Grade C encompasses trials with notable flaws or a high risk of bias, in addition to case reports or case series. Medication sequences classified as Grade C should be cautiously considered and require a comprehensive analysis in conjunction with the specific treatment context.

This study employs the Micromedex Thomson^®^ grading system as a reference, incorporating the quality of evidence collected and team discussions, to categorize medication sequences into three grades:

#### 2.5.1 Grade A

Grade A evidence primarily encompasses two aspects: Firstly, randomized controlled trials (RCTs) and meta-analyses that integrate findings from multiple independent high-quality studies, along with well-designed randomized clinical trials. Secondly, explicit drug administration sequences specified in official package inserts or drug registration documents. These specifications are directly classified as Grade A evidence because they represent authoritative recognition by regulatory agencies, are based on extensive research and clinical practice, and exhibit high reliability and safety. Grade A evidence is characterized by the rationality of its study design, with a low risk of bias upon evaluation. Additionally, it provides detailed and transparent data to support statistical analysis and validation, enabling the integration of data from multiple studies through quantitative analysis. Utilizing confidence intervals and p-values, Grade A evidence demonstrates the reliability and reproducibility of its conclusions. Additionally, it adheres to principles governing medication sequencing, such as cell proliferation kinetics, drug interactions, chronopharmacology, and drug irritability. Therefore, we recommend adopting medication sequences based on Grade A evidence in clinical practice.

#### 2.5.2 Grade B

Grade B evidence primarily encompasses meta-analyses of randomized controlled trials (RCTs) with conflicting conclusions, RCTs of small scale or with partial methodological flaws, and non-randomized studies. The characteristics of Grade B evidence are that they generally align with medication administration experiences and patterns, yet they are mostly based on small-scale studies. These studies tend to have relatively small sample sizes, and these studies may show conflicts with each other or with established drug sequencing principles. Due to the limited number of studies precludes effective pooling and quantitative analysis, thereby constraining the generalizability and reliability of the results. Additionally, some studies exhibit certain flaws, potentially due to limitations in study design, such as inadequate randomization, incomplete data, or inappropriate analytical methods, which may adversely affect the reliability of the results. Despite the lack of extensive validation and robust evidence, given the scarcity of relevant research, we still recommend appropriately referencing the medication administration sequences supported by Grade B evidence.

#### 2.5.3 Grade C

Grade C evidence mainly includes trials with significant flaws or high risk of bias, case reports, or series of cases. The characteristic of Grade C evidence is the presence of studies with obvious defects, which may have major flaws or a high risk of bias in their design, implementation, or analysis processes. Due to the scarcity of literature, effective pooling and quantitative analysis are also not possible, limiting the generalizability and reliability of the results. These studies can only serve as references and cannot be used as the main basis for decision-making. Additionally, they have an extremely small sample size or lack a control group, and their findings may contradict principles governing medication sequencing, the results await further verification. Therefore, given the relatively low quality and reliability of Grade C evidence, its utilization necessitates cautious analysis within the specific therapeutic context.

## 3 Results

**Figure 1:**
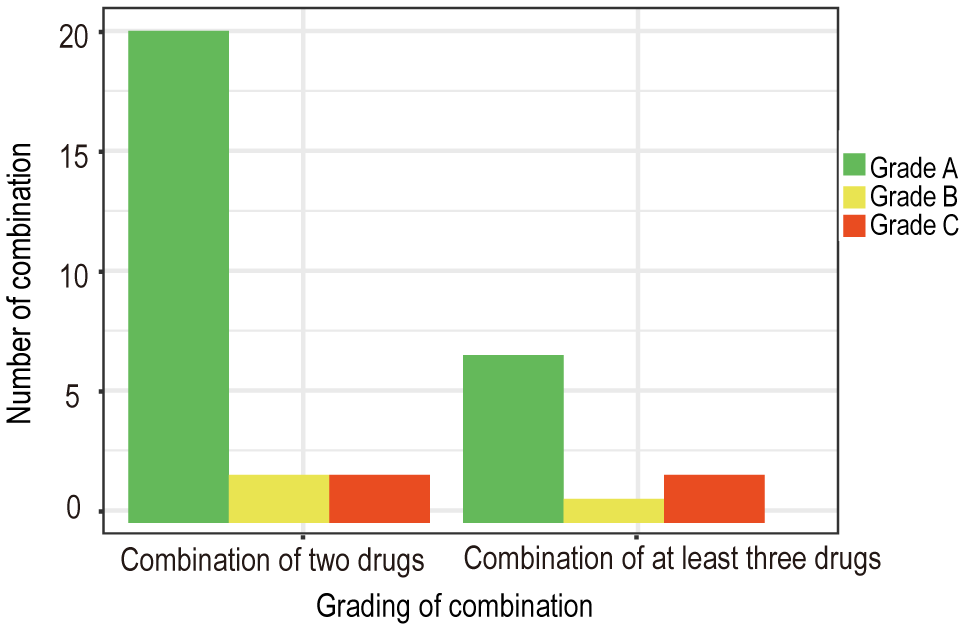
Quantity and grading of combined chemotherapy regimens for breast cancer.

Based on the collected data, we systematically categorized and organized the information. Subsequently, adhering to the aforementioned evidence-based grading criteria and incorporating the comprehensive analysis of our research team, we classified and recommended the conventional drug administration sequences within 24 hours (see **Tables 1**, **2**). According to our statistical findings, we identified 24 common two-drug combinations used in breast cancer chemotherapy regimens (Grade A: 20, Grade B: 2, Grade C: 2), along with 10 combinations involving three or more drugs (Grade A: 7, Grade B: 1, Grade C: 2), as detailed in **Figure 1**.

**Table 1:** Recommended sequencing and evidence-based grades for dual-drug combinations.

Drug Regimen		Step1	Step2	Analysis Findings	Reference	Evidence Grade
AC EC	A 60mg/m^2^ iv E 90~100mg/m^2^ iv C 600~830mg/m^2^ iv	C	A/E	Effectiveness↑ Irritation↓	(22)	C
AT ET	A 50mg/m² iv E 60~75mg/m^2^ iv T 75mg/m^2^ iv	A/E	T	Effectiveness↑ Toxicity↓	(18, 23–26)	A
GT	G 1000~1250mg/m^2^ iv T 75/175mg/m^2^ iv	T	G	Toxicity↓	(22, 27, 28)	A
GCb GP	G 1000~1250mg/m^2^ iv Cb AUC=6 iv P 75mg/m^2^ iv	G	Cb/P	Toxicity↓	(22, 29–31)	A
HX^*^	H 6/8mg/kg iv X 1000mg/m^2^ po	H	X	No significant impact	(32, 33)	A
HT	H 2/4/6mg/kg iv T 75/100mg/m2 iv	H	T	Effectiveness↑ Toxicity↓	(22, 34–37)	A
L^*^X^*^	L 1250mg po X 1000mg/m^2^ po	L/X	X/L	No significant impact	(32, 38)	A
L^*^H	L 1000mg po H 2/4/6/8mg/kg iv	H	L	No significant impact	(33, 38)	A
NH	N 25 mg/m^2^ iv H 2/4mg/kg iv	H	N	No significant impact	(33, 36)	A
NI	N 25mg/m^2^ iv I 2/4/6/8mg/kg iv	I	N	No significant impact	(36, 39)	A
NX^*^	N 25mg/m^2^ iv X 950~1000mg/m^2^ po	N/X	X/N	No significant impact	(32, 36, 40, 41)	A
NCb NP	N 25mg/m^2^ iv Cb AUC=6 iv P 75 mg/m^2^ iv	Cb/P	N	Effectiveness↑	(42–44)	B
Py^*^X^*^	Py 400mg/qd po X 950~1000mg/m^2^ po	Py+X	X	No significant impact	(32, 45, 46)	A
TP	T 75/175mg/m^2^ iv P 75 mg/m^2^ iv	T	P	Toxicity↓	(22, 47)	A
TX^*^	T 75mg/m^2^ iv X 950~1000mg/m^2^ po	T/X	X/T	No significant impact	(32)	A
TCb	T 75~100mg/m^2^ iv Cb AUC=6 iv	T	Cb	Effectiveness↑ Toxicity↓	(48–50)	A
TC	T 75mg/m^2^ iv C 600mg/m^2^ iv	C	T	Effectiveness↑ Toxicity↓	(22, 51)	A
T+B	T 75mg/m^2^ iv B 10~15 mg/kg iv	T	B	B in step 2 for the first time	(28)	A
UX^*^	U 30 mg/m² iv X 1250mg/m² po	U/X	X/U	No significant impact	(52, 53)	A
X^*^B	X 1250mg/m² po B 10~15mg/kg iv	X	B	B in step 2 for the first time	(28, 32)	A

↑ indicates increased effectiveness.↓↓ indicates reduced toxicity or irritation.

**Table 2:** Recommended sequencing and evidence-based grades for triple-drug or more combinations.

Drug Regimen		Step1	Step2	Step3	Analysis Findings	Reference	Evidence Grade
C^*^MF	C 100mg/m^2^ po M 40mg/m^2^ iv F 600mg/m^2^ iv	C	M	F	Effectiveness↑	(22, 50, 54)	A
FAC FEC	F 500mg/m^2^ iv A 50mg/m^2^ iv C 500mg/m^2^ iv	C	A/E	F	Effectiveness↑	(22)	C
TAC	T 75mg/m^2^ iv A 50mg/m^2^ iv C 500mg/m^2^ iv	C	A	T	Effectiveness↑ Toxicity↓	(22, 25, 51, 55–58)	B
TX^*^H	T75mg/m^2^ iv X 950~1000mg/m^2^ po H 2/4mg/kg iv	H	T/X	X/T	Effectiveness↑ Toxicity↓	(32, 33, 37)	A
THPa	T 75mg/m^2^ iv H 2/4mg/kg iv Pa 420/840mg iv	H/Pa	Pa/H	T	Effectiveness↑ Toxicity↓	(22, 33, 37, 59)	A
TCbHPa	T 75~100mg/m^2^ iv Cb AUC=6 iv H 2/4mg/kg iv Pa 420/840mg iv	H/Pa	T	Cb	Toxicity↓	(33, 37, 49, 60)	A
KTCb	K 200mg/m^2^ iv T 75mg/m^2^ iv Cb AUC=6 iv	K	T	Cb	Effectiveness↑ Toxicity↓	(49, 59, 61)	A
KAC KEC	K 200mg/m^2^ iv A 60mg/m^2^ iv C 600mg/m^2^ iv	K	C	A/E	Toxicity↓	(59, 61)	A

The asterisk (*) in the upper right corner indicates oral administration route.

A, Doxorubicin; B, Bevacizumab; C, Cyclophosphamide; Cb, Carboplatin; E, Epirubicin; F, Fluorouracil; G, Gemcitabine; H, Trastuzumab; L, Lapatinib; K, Pembrolizumab; M, Methotrexate; N, Vinorelbine; P, Cisplatin; Pa, Pertuzumab; Py, Pyrotinib; T, Paclitaxel/Docetaxel; U, utidelone; I, Inetetamab; X, Capecitabine.

↑ indicates increased effectiveness.↓↓ indicates reduced toxicity or irritation.

To further evaluate the efficacy of these chemotherapy regimens, we excluded drug package inserts and specialized book literature from the Grade A sources and extracted adverse reaction data from eight trials. Using Stata 16 software, we conducted statistical analyses on two groups: the recommended drug sequencing group and the control group. Quantitative analysis revealed an I² heterogeneity of 0, indicating minimal variation among the studies. The pooled odds ratio (OR) was 0.67, with a 95% confidence interval of (0.52, 0.85), and a P-value of 0.002, demonstrating a statistically significant difference. Furthermore, Begg’s test was performed to assess the risk of bias in the studies, yielding a P-value of 0.322 and an adjusted P-value of 0.386, both greater than 0.05, indicating no significant risk of bias was detected. These results underscore the effectiveness of rational chemotherapy sequencing in reducing the incidence of adverse reactions.

For Grade B and C chemotherapy sequences, due to the limited number of relevant studies and the inability to effectively pool the data, we conducted only qualitative analyses. Detailed analysis results are presented in **Tables 1**, **2**, and the Discussion section.

### 3.1 Analysis of ‘no significant impact’

Based on the data we have collected and analyzed, a total of nine combination medication regimens have been identified that do not show significant impact from the order of administration. Our conclusion is primarily based on the following considerations: Firstly, through extensive review of literature and pharmaceutical data, we found no significant adverse reactions or specific usage requirements associated with the order of these drug combinations; secondly, these combinations are predominantly common practice in both pharmaceutical registration trials and clinical settings, possessing substantial practical experience, and currently, there is no evidence suggesting differences in safety or efficacy arising from variations in the administration sequence. Therefore, we have classified these regimens as “having no major impact,” meaning that the order of administration does not significantly matter, and after conducting a multifaceted rationality analysis, we have categorized these regimens as Grade A, indicating high safety in combined therapy.

However, given the limited direct research on administration sequence and the demands of medical practice, we are still attempting to conduct a comprehensive analysis of the rationality behind drug administration sequences from multiple perspectives. This analysis encompasses key factors such as cell proliferation kinetics, drug interactions, chronopharmacology, drug irritancy, and clinical medication experiences.

#### 3.1.1 Combination therapy with monoclonal antibodies

The combinations of HN, HX, and IN involve monoclonal antibodies. After consulting relevant literature, it was found that there is no pharmacokinetic impact on the drug administration sequence for these regimens. These sequences are recommended in the drug registration documents. Monoclonal antibody drugs generally exhibit stable pharmacokinetics, demonstrating a high level of safety and efficacy across diverse populations (62–66). Typically, drug labels recommend initiating the infusion with monoclonal antibody drugs as the first step, allowing for better observation of patient tolerance and adverse reactions (67). considering the therapeutic benefits observed in other combination therapies involving monoclonal antibodies (61), in the absence of high-grade evidence, we recommend prioritizing the administration of monoclonal antibody drugs.

#### 3.1.2 Combination therapy with capecitabine

The combinations of XT, XN, PyX, and XU involve capecitabine. Capecitabine inhibits CYP2C9, potentially increasing adverse reactions when co-administered with drugs metabolized through the CYP2C9 pathway (32). However, in commonly used combination therapies for breast cancer, no evidence of toxic interactions has been identified. There is also no evidence of pharmacokinetic interactions between capecitabine and paclitaxel or docetaxel (68).

Additionally, studies indicate that the accumulation ratio of area under the curve (AUC) for capecitabine when co-administered with pyrotinib is approximately 1, suggesting no significant drug accumulation (46). Considering pharmacokinetic absorption, we recommend oral administration with water within 30 minutes after meals, twice daily with a 12-hour interval. Capecitabine exhibits significant circadian rhythm variations in pharmacokinetics. Administering capecitabine in the morning and evening respectively can achieve high AUC levels of 5-fluorouracil and better treatment responses (69). To enhance patient compliance and improve drug efficacy by reducing the dosing frequency, we recommend co-administering pyrotinib and capecitabine in the morning with breakfast for the first dose, and administering the second dose of capecitabine in the evening with dinner.

#### 3.1.3 Combination therapy with lapatinib

Combinations HL and XL involve lapatinib. According to the U.S.FDA prescribing information and related data, pyrotinib inhibits CYP3A4, CYP2C8, and P-glycoprotein (P-gp, ABCB1) at clinically relevant concentrations *in vitro*. It is also a weak inhibitor of CYP3A4 *in vivo*, and this interference does not overlap with the main metabolic pathways of capecitabine and trastuzumab (32, 33, 38, 70). Multiple studies have demonstrated the safety and efficacy of combinations HL and XL (71–73). Therefore, administration according to the conventional usage is appropriate. However, considering the common adverse reactions (diarrhea, nausea, vomiting, rash, etc.) associated with the three drugs (74–77), it may be beneficial to extend the interval between the two drugs. For example, we recommend administering trastuzumab in the morning and lapatinib one hour after dinner (once daily at a fixed time), thereby reducing the potential occurrence of adverse reactions.

### 3.2 Evidence analysis for grades B and C

In our analysis of the collected Level B and C evidence, we have noticed preliminary studies conducted on these drug administration sequences. However, the evidence remains relatively scarce, leading to uncertainty in the conclusions. Adhering to the classification criteria outlined previously, we have categorized this evidence as either Level B or C. Additionally, we have conducted a further analysis to determine the rational drug administration sequence.

#### 3.2.1 Combination therapy with cyclophosphamide

AC, EC, FAC, FEC, TAC. Cyclophosphamide can be administered orally or intravenously. Both animal and human experiments have shown no significant differences in the pharmacokinetics, safety, and efficacy of oral and intravenous administration of cyclophosphamide (78–81). Cyclophosphamide is activated after metabolism by CYP2B6 and CYP2C9, and it does not interact with other drugs in the mentioned regimens. Considering circadian drug efficacy and principles of cell proliferation kinetics, there is literature recommending the administration of cyclophosphamide in the morning (22). Combining these principles with clinical practice, we recommend initiating the medication regimen with the use of cyclophosphamide as the first step.

Referring to the U.S.FDA prescribing information for cyclophosphamide, it indicates potential pharmacodynamic or pharmacokinetic interactions when combined or sequentially administered with other drugs. Ethanol may reduce the antitumor activity of cyclophosphamide (51), and both paclitaxel and docetaxel contain ethanol (except for paclitaxel liposome and albumin-bound paclitaxel). Moreover, administering cyclophosphamide after paclitaxel infusion can increase hematologic toxicity. Considering these factors, it is advisable to extend the interval between the administration of cyclophosphamide and paclitaxel. In the TAC regimen, we recommend the infusion sequence as C, A, T. Additionally, in a pharmacokinetic study on the FEC regimen for breast cancer patients, no clinically significant correlations were found among the drugs in different compartment models (82). Considering the principles of cell proliferation kinetics, we recommend the administration sequence as C, A/E, F.

#### 3.2.2 Combination therapy with vinorelbine

Including NCb and NP. Clinical trial data directly comparing drug administration sequences in this regimen are lacking. Studies have suggested that administering cisplatin first, followed by sequential infusion of vinorelbine, is well-tolerated, yielding encouraging results in terms of response rate (RR), median time to progression (TTP), and overall survival (OS) (42–44). Additionally, a phase II trial has assessed the comparability of safety and tolerability between carboplatin and cisplatin (83). Considering the principles of breast cancer cell proliferation kinetics, we recommend utilizing the cell cycle non-specific agents cisplatin/carboplatin before vinorelbine.

## 4 Discussion

When selecting the sequence of combined drug administration, we often encounter situations where there is a lack of evidence from evidence-based medicine or conflicting results from different studies. In the context of choosing the drug administration sequence, we primarily consider the following aspects.

### 4.1 Proliferation kinetics

Different types of tumor cells have varying growth cycles, and for proliferative breast cancer, the average doubling time of tumor cells is 150 days (much shorter for Burkitt lymphoma and acute lymphocytic leukemia, with a doubling time of less than 5 days), indicating a relatively slow-growing tumor (22, 84–86). In the treatment strategy for breast cancer, it is common to initially administer cell cycle non-specific agents (CCNSA) to significantly eliminate tumor cells, reducing their overall number. This process prompts more G0 phase cells to enter the proliferative phase. Subsequently, cell cycle-specific agents (CCSA) are administered to target cells re-entering the proliferative cycle, achieving optimal therapeutic effects(Yuanying 54, 87). The action cell cycles of commonly used drugs for breast cancer are outlined in **Table 3**.

**Table 3:** Common drugs targeting the proliferation cycle of tumor cells in breast cancer.

Pharmacological category	Cycle-targeted	Medication Name
CCNSA	Entire cycle	Doxorubicin Cyclophosphamide Carboplatin Epirubicin Cisplatin
CCSA	G1 phase	Trastuzumab Lapatinib Pertuzumab Pyrotinib
	S phase	Fluorouracil Gemcitabine Methotrexate Capecitabine
	Late G2 phase	Paclitaxel Docetaxel
	M phase	Vinorelbine Paclitaxel Docetaxel Utidelone

Drugs Bevacizumab, Pembrolizumab, and Inetetamab have been excluded from the table due to the lack of direct modulation on the cell proliferation cycle.

### 4.2 Drug interactions

Drug interactions involve both pharmacokinetic factors (mainly affecting absorption, distribution, metabolism, and excretion) and pharmacodynamic factors (including unrelated, synergistic, additive, and antagonistic effects on efficacy) (88). Due to the narrow therapeutic index and limited safety range of anticancer drugs, even subtle changes in the pharmacokinetic and pharmacodynamic characteristics caused by drug interactions can significantly alter the toxicity or efficacy of drugs (89). Therefore, the potential impact of drug interactions in cancer therapy should be widely recognized.

The pharmacodynamics and pharmacokinetics of drug interactions involve various influencing factors, including dosage, administration route, half-life, steady-state blood concentration time, hepatic extraction ratio, elimination pathway, etc. Additionally, many anticancer drugs are inhibitors of cytochrome P450 isoenzymes, and some drugs are metabolized by these enzymes. Thus, extensive interactions exist among anticancer drugs, easily affecting drug efficacy (90). For example, in combination chemotherapy regimens containing taxanes, taxanes are generally administered first, while platinum agents have some nephrotoxicity and are usually administered later. Especially when cisplatin is used in combination with taxanes, the correct administration sequence is crucial, as cisplatin can decrease the clearance rate of paclitaxel. If the proper administration sequence is not followed, it can increase the toxicity of the chemotherapy regimen (22). Similarly, monoclonal antibody drugs are typically administered before chemotherapy drugs (36). On the one hand, any infusion-related adverse reactions from monoclonal antibodies are more easily detected. On the other hand, the anti-angiogenic effects of monoclonal antibodies synergistically enhance the subsequent chemotherapy drugs’ efficacy (91, 92). Therefore, it is recommended to administer monoclonal antibody drugs first.

### 4.3 Chronopharmacology

Currently, studies have identified more than fifty anticancer drugs with time-dependent efficacy and toxicity (93). Various tumor types display time-dependent sensitivity to chemotherapy drugs, implying that administering the same drug dose at different times throughout the day may lead to variations in the extent of tumor cell eradication. Furthermore, the body’s tolerance to chemotherapy drug toxicity and the drug disposition process (absorption, distribution, metabolism, excretion) also varies over time (94–97).

Research indicates that selecting an appropriate circadian rhythm for drug administration significantly improves treatment outcomes, reducing drug toxicity by about five times and enhancing antitumor efficacy by nearly two times (93, 95). Taking fluorouracil as an example, its plasma concentration shows significant circadian variation during constant-rate intravenous administration. This variation may be related to the circadian rhythmicity of the key enzyme DPD (dihydropyrimidine dehydrogenase), which exhibits higher activity from midnight to around 4 am, reaching its peak. At this time, the body can tolerate higher doses with lower drug toxicity (98). Similar metabolic rhythms have been observed in studies involving gemcitabine as well (99). Practice has shown that aligning drug administration with the pharmacological characteristics of the drugs based on circadian rhythms can achieve better efficacy and lower toxicity (100, 101). For instance, platinum-based drugs, such as cisplatin, may benefit from nighttime administration, as their protein binding rate is highest around 4 pm, maintaining high concentrations of the drug for an extended period, resulting in better efficacy (36, 50). Literature also indicates that maximizing methotrexate toxicity occurs when administered at 6 am, whereas minimizing toxicity is achieved when administered at midnight (102). Another study indicates that nighttime administration can improve the therapeutic index of docetaxel, reducing side effects (103). Some drugs are better suited for daytime administration, such as Doxorubicin, which exhibits one-third lower toxicity when administered at 8 am compared to 8 pm (102), and cyclophosphamide exhibits the most sustained and optimal tolerance when administered between 10 am and 2 pm. Additionally, administering cyclophosphamide first aligns with the principles of cell proliferation kinetics. These are the reasons why we consider administering cyclophosphamide as the initial infusion in the treatment plan (104, 105).

It is important to note that, whether for outpatient visits or hospitalized patients, chemotherapy drugs are usually administered in the morning, primarily due to considerations of hospital workflow continuity and patient convenience during visits. However, even with the knowledge of the ideal drug administration timing, conflicts may arise with pharmacy drug distribution, clinic opening hours, and patient’s nighttime rest period. Therefore, in clinical practice, more flexible administration methods, such as novel oral pulse administration or intravenous drug pumps, can be employed. Chronopharmacology requires simultaneous consideration of various factors. The administration sequence and timing should align with the biological rhythms of chronopharmacology, taking into account both the pharmacokinetic characteristics of anticancer drugs and the specific conditions of individual patients to select the optimal timing for drug administration.

### 4.4 Drug irritancy

Chemotherapeutic drugs can be categorized based on their irritancy into vesicant and irritant drugs (**Table 4**). Vesicant drugs are those that, when extravasated from the vascular route into surrounding tissues, can cause blistering, tissue necrosis, or decay. Vesicant agents typically elicit sensations of burning or stinging upon contact with the body. On the other hand, irritant drugs cause local tissue burns, irritation, or mild inflammation after extravasation but do not directly lead to tissue necrosis (106).

**Table 4:** Classification of chemotherapy drugs for breast cancer based on irritant magnitude.

Irritancy category	Drug category	Medication Name
irritative drugs	Alkylating agent	Cyclophosphamide
	Antibiotics	Doxorubicin-liposome
	Antimetabolites	Fluorouracil Methotrexate Gemcitabine
	Platinum-based	Cisplatin Carboplatin
vesicant drugs	Antibiotics	Doxorubicin Epirubicin
	Alkaloids	Vinorelbine
	Taxanes	Paclitaxel Paclitaxel (albumin-bound) Docetaxel
	Platinum-based	Cisplatin(high concentrations)

When using two or more anticancer drugs and lacking specific instructions in the drug manual regarding the administration sequence, consideration is given to the irritative nature of the drugs. Some literature suggests that in combined chemotherapy, it is advisable to administer less vascularly irritating drugs first, followed by more irritating vesicant drugs. The rationale behind this approach is to facilitate patient adaptation to the infusion process, thereby improving treatment compliance (107).

However, other studies propose an alternative perspective, advocating the initiation of chemotherapy with vesicant drugs. The reasoning is that at the beginning of chemotherapy, the venous structure is most stable, reducing the chances of drug extravasation. Additionally, less irritative drugs used subsequently can help flush the venous walls effectively (17, 36, 106). It is also noted that, based on the extent of tissue damage caused by extravasation of chemotherapy drugs, administering vesicant drugs first, followed by non-vesicant drugs, is recommended. If both drugs are vesicant, it is suggested to administer the one with a higher concentration first (108). While we lean towards the latter viewpoint, considering the lack of in-depth research and high-quality evidence on the sequence related to irritative drug, we recommend a comprehensive consideration of other medication principles, relevant evidence, and individual patient compliance factors.

### 4.5 Hierarchy of clinical evidence

International consensus, drug package insert, clinical practice guidelines, and consensus statements are internationally recognized as Grade A evidence (109). We rely on these sources as much as possible in collecting relevant information. However, considering the limited quantity of relevant trials and evidence on drug administration sequences, if there is a lack of evidence-based data on the administration sequence, the results of drug administration sequences in large-scale clinical randomized studies, validated for safety and efficacy, are also considered as crucial factors in our considerations.

## 5 Practice and obstacles

In clinical practice, we recommend updating the existing prescription management systems to enable automatic analysis of prescribed chemotherapy regimens, automatically generating rational drug administration schedules, and clearly indicating the sequence on electronic prescriptions and drug labels. Additionally, the system should provide detailed instructions on drug administration sequences, allowing physicians to make more scientific and rational medication arrangements after comprehensively considering the patient’s specific conditions.

However, the obstacles to be overcome in practical applications cannot be ignored, and they primarily encompass three aspects: Firstly, the practice of individualized drug administration necessitates adhering to optimized sequences while fully considering individual patient differences, disease progression, and treatment preferences. This requires establishing a more refined and easily operable individualized assessment pathway, such as scoring and grading patients before treatment (110, 111). Secondly, the challenge of multi-team collaboration ensures that every step, from prescription writing to drug preparation to administration, strictly follows the optimized sequence. Medical institutions must establish effective communication mechanisms and execution supervision systems to avoid sequence errors caused by premature preparation or arbitrary drug administration. Thirdly, continuous evidence-based practice is essential due to the limitations of current research data. Ongoing clinical research should be promoted, and methods such as metabolomics, network pharmacology, and disease treatment network analysis should be fully utilized to continuously enrich and update the existing evidence base, providing a more solid foundation for optimizing chemotherapy sequences (112–115).

## 6 Limitations

In the first place, this study exclusively analyzes the drug administration sequence of commonly used combination chemotherapy regimens for breast cancer and cannot directly apply the conclusions to other types of tumors. Secondly, in the collection and evaluation of evidence-based data, it was observed that a small portion of regimens lacked direct comparative data on the superiority or inferiority of drug administration sequences. In such cases, inference and analysis relied on principles such as pharmacokinetics, pharmacodynamics, and cell proliferation kinetics, introducing potential biases and uncertainties.

Lastly, when providing recommendations for drug administration sequences, this study did not take into account individual factors among different patients, such as liver and kidney function, drug allergy history and concurrent medication. These factors may influence drug metabolism, distribution, excretion, and effects, leading to variations in drug administration sequences.

## 7 Conclusion

Considering the complexity of oncology drug treatments and clinical practices, the mechanism of action for the same combination chemotherapy regimen may not be entirely consistent across different cancer treatments. This variation can lead to different conclusions regarding drug administration sequences in various literature. Therefore, in clinical practice, we recommend prioritizing sequential drug administration according to the official drug product labeling. Simultaneously, it is essential to consider factors such as the administration sequence from large randomized controlled trials, the cell proliferation kinetics of the specific cancer type, drug interactions, chronopharmacology, drug irritability, clinical experiences, and patient preferences. By taking these factors into account, we aim to maximize treatment efficacy while minimizing the occurrence of adverse reactions.

## Data Availability

The original contributions presented in the study are included in the article/**Supplementary Material**. Further inquiries can be directed to the corresponding author.
