# Supplementary material for: Administration sequences in single-day chemotherapy regimens for breast cancer: a comprehensive review from a practical perspective
Source: Front Oncol. 2024 Sep 30;14:1353067. doi: 10.3389/fonc.2024.1353067 (PMC11471725; doi:10.3389/fonc.2024.1353067)
Supplement: Supplementary file 1 [file Table1.docx]

**Search strategies**

| Literature databases | Search items | Items found |
| --- | --- | --- |
| (1) PubMed | Search: ((chemotherapy[Title/Abstract]) OR (Chemotherapeutics[Title/Abstract]) OR (Chemotherapies[Title/Abstract]) OR (Chemo[Title/Abstract]) OR (Cytotoxic chemotherapy[Title/Abstract]) OR (Drug[Title/Abstract]) OR (Pharmacotherapy[Title/Abstract]) OR (Cytotoxic therapy[Title/Abstract]) OR (Anticancer drug therapy[Title/Abstract]) OR (Drug therapy[Title/Abstract]) OR (Chemical therapy[Title/Abstract])) AND ((sequence) OR (Order) OR (Series) OR (Succession) OR (Chain) OR (String) OR (Arrangement) OR (Concatenation) OR (Array) OR (Queue) OR (Row) OR (Concatenation) OR (Continuance) OR (Continuity) OR (Ordering) OR (Immediately following) OR (Right after) OR (Directly after) OR (Subsequent to) OR (Closely following) OR (after) OR (Following closely) OR (Post) OR (In the next instant)) AND ((24 hours) OR (period) OR (day) OR (Around the clock) OR (Day and night) OR (Continuous day) OR (Complete day) OR (Whole day) OR (Full day) OR (All day long) OR (Throughout the day) OR (Without interruption) OR (Phase) OR (Duration) OR (Short-term)) | 7074 |
| (2)Web of science | (TS=(chemotherapy) OR TS=(Chemotherapeutics) OR TS=(Chemotherapies) OR TS=(Cytotoxic chemotherapy) OR TS=(Drug regimen) OR TS=(Pharmacotherapy) OR TS=(Cytotoxic therapy) OR TS=(Anticancer drug) OR TS=(Chemical therapy)) AND (TS=(sequence) OR TS=(Arrangement) OR TS=(Concatenation) OR TS=(Array) OR TS=(Concatenation) OR TS=(Continuity) OR TS=(Immediately following) OR TS=(Right after) OR TS=(Directly after) OR TS=(Subsequent to) OR TS=(Closely following) OR TS=(Following closely) OR TS=(next instant)) AND (((TS=(24 hours) OR TS=(full day) OR TS=(single day) OR TS=(Continuous)) OR TS=(Complete day) OR TS=(Without interruption) OR TS=(Duration)) OR TS=(Short-term)) | 6108 |
| (3)the Cochrane Library | ((chemotherapy) OR (Chemotherapeutics) OR (Chemotherapies) OR (Chemo) OR (Cytotoxic chemotherapy) OR (Drug) OR (Pharmacotherapy) OR (Cytotoxic therapy) OR (Anticancer drug therapy) OR (Drug therapy) OR (Chemical therapy)) in Title Abstract Keyword AND ((sequence) OR (Order) OR (Series) OR (Succession) OR (Chain) OR (String) OR (Arrangement) OR (Concatenation) OR (Array) OR (Queue) OR (Row) OR (Concatenation) OR (Continuance) OR (Continuity) OR (Ordering) OR (Immediately following) OR (Right after) OR (Directly after) OR (Subsequent to) OR (Closely following) OR (after) OR (Following closely) OR (Post) OR (In the next instant)) in Title Abstract Keyword AND ((24 hours) OR (period) OR (day) OR (Around the clock) OR (Day and night) OR (Continuous day) OR (Complete day) OR (Whole day) OR (Full day) OR (All day long) OR (Throughout the day) OR (Without interruption) OR (Phase) OR (Duration) OR (Short-term)) in Title Abstract Keyword | 2473 |
| (4)Embase | ((chemotherapy) OR (Cytotoxic) OR (Anticancer) OR (regime) OR (drug)) AND ((sequence) OR (Order)) AND ((24 hours) OR (single day)) | 1392 |
| (5)China National Knowledge Infrastructure Database | (主题: 化疗 + 向治疗 + 药物 + 化学治疗 + 肿瘤) AND (主题: 序顺序 + 先后 + 联合) AND (主题: 单日 + 一日 + 同日 + 24小时) | 940 |
| (6)Wan Fang database Search strategy | 主题:(("化疗" or "靶向治疗" or " 药物" or "化学治疗" or "抗肿瘤药")) and 主题:(("序贯" or "顺序" or "先后" or "联合")) and 主题:(("单日" or "一日" or "同日" or "24小时")) | 156 |
| Overall | / | 18143 |
