# Supplementary material for: Administration sequences in single-day chemotherapy regimens for breast cancer: a comprehensive review from a practical perspective
Source: Front Oncol. 2024 Sep 30;14:1353067. doi: 10.3389/fonc.2024.1353067 (PMC11471725; doi:10.3389/fonc.2024.1353067)
Supplement: Supplementary file 2 [file Table2.docx]

Additional records added manually from other sources

(n = 17)

Records identified through databases searching

(n = 18,143)

**Identification**

Reports assessed for eligibility

(n = 238)

Assessing relevance through abstracts (n = 12,193)

Records screened

(n = 18,160)

Duplicates excluded

(n = 5,967)

**Screening**

Non-relevant abstracts excluded

(n = 11,955)

**Eligibility**

Full-text articles excluded with reasons (n=198)

1. No relevant outcomes(n=157)

2. Incomplete data(n=23)

3. No comparator or inappropriate comparisons(n=18)

Studies included in the systematic review (n = 40)

**Included**
